# Radiomic and dosimetric parameter-based nomogram predicts radiation esophagitis in patients with non-small cell lung cancer undergoing combined immunotherapy and radiotherapy

**DOI:** 10.3389/fonc.2024.1490348

**Published:** 2024-12-18

**Authors:** Kang Wang, Junfeng Zhao, Jinghao Duan, Changxing Feng, Ying Li, Li Li, Shuanghu Yuan

**Affiliations:** ^1^ Department of Radiation Oncology, Shandong Cancer Hospital and Institute, Shandong First Medical University, and Shandong Academy of Medical Sciences, Jinan, Shandong, China; ^2^ Department of Medical Oncology, Shandong Cancer Hospital and Institute, Shandong First Medical University, and Shandong Academy of Medical Sciences, Jinan, Shandong, China; ^3^ Department of Radiation Oncology, The First Affiliated Hospital of University of Science and Technology of China (USTC), Division of Life Sciences and Medicine, University of Science and Technology of China, Hefei, Anhui, China; ^4^ Department of Radiation Oncology, Anhui Provincial Cancer Hospital, Hefei, Anhui, China

**Keywords:** radiation esophagitis, non-small-cell lung cancer, radiomics, radiotherapy, immunotherapy

## Abstract

**Background:**

The combination of immune checkpoint inhibitors (ICIs) and radiotherapy (RT) may increase the risk of radiation esophagitis (RE). This study aimed to establish and validate a new nomogram to predict RE in patients with non-small cell lung cancer (NSCLC) undergoing immunochemotherapy followed by RT (ICI-RT).

**Methods:**

The 102 eligible patients with NSCLC treated with ICI-RT were divided into training (n = 71) and validation (n = 31) cohorts. Clinicopathologic features, dosimetric parameters, inflammatory markers, and radiomic score (Rad-score) were included in the univariate logistic regression analysis, and factors with *p* < 0.05 in the univariate analysis were included in the multivariate logistic regression analysis. Factors with significant predictive values were obtained and used for developing the nomogram. The area under the receiver operating characteristic curve (AUC), calibration curve, and decision curve were used to validate the model.

**Results:**

A total of 38 (37.3%) patients developed RE. Univariate and multivariate analyses identified the following independent predictors of RE: a maximum dose delivered to the esophagus >58.4 Gy, a mean esophagus dose >13.3 Gy, and the Rad-score. The AUCs of the nomogram in the training and validation cohorts were 0.918 (95% confidence interval [CI]: 0.824–1.000) and 0.833 (95% CI: 0.697–0.969), respectively, indicating good discrimination. The calibration curves showed good agreement between the predicted occurrence of RE and the actual observations. The decision curve showed a satisfactory positive net benefit at most threshold probabilities, suggesting a good clinical effect.

**Conclusions:**

We developed and validated a nomogram based on imaging histological features and RT dosimetric parameters. This model can effectively predict the occurrence of RE in patients with NSCLC treated using ICI-RT.

## 1 Introduction

Lung cancer is the leading cause of cancer-related death globally, with non-small cell lung cancer (NSCLC) accounting for approximately 85% of all lung cancers. Approximately one-third of patients with NSCLC have locally advanced disease at the time of diagnosis (1). The emergence of immune checkpoint inhibitors (ICIs) has transformed the treatment of NSCLC, bringing new options for clinical care with significant efficacy in improving disease control, overall survival, and quality of life (2, 3). Radiotherapy (RT) has long been a cornerstone of NSCLC treatment (4). The combination of ICIs and RT has a synergistic effect on NSCLC and can enhance the tumor-killing effect of effector T cells and the distant effect of RT (5, 6).

The side effects of RT remain a significant challenge for treatment management. Radiation esophagitis (RE) usually occurs 2–4 weeks after the start of treatment, and some symptoms, such as progressive dysphagia, can appear up to 2 months after RT. Despite advances in RT techniques, RE remains one of the major toxicities among patients with NSCLC following RT (7). The incidence of RE ≥grade 2 ranges from 30% to 50% and increases at higher radiation doses (8). RE can lead to dysphagia, retrosternal pain, and even esophageal ulcer or fistula formation in severe cases. The development of RE affects the quality of life of the patient and may also require interruption or early termination of treatment, resulting in a significant financial burden and poor prognosis (8, 9). Importantly, RE affects local tumor control, and severe RE has a negative impact on overall survival (10, 11). Therefore, early identification of patients with risk factors for developing RE allows clinicians to take appropriate preventive measures, such as pharmacologic prophylaxis, dietary guidance, or nasal feeding. Identifying patients at low risk for RE provides the opportunity to moderately increase the dose of RT to improve tumor control.

Using radiomics, medical images can be converted into mineable data through high-throughput extraction of quantitative features, which is promising for cancer diagnosis, prognosis, and treatment-response prediction (12–14). The maximum dose delivered to the esophagus (D_max_), mean esophagus dose (MED), percent of esophagus volume receiving ≥50 Gy (V_50_), concurrent chemoradiotherapy (cCRT), neutrophil nadir during RT, high platelet counts, and low hemoglobin levels before treatment have been associated with the development of RE (15, 16).

However, the above studies have focused on the high-risk factors for RE in patients with NSCLC undergoing cCRT, and did not analyze the data of patients treated with ICIs prior to RT. In this study, we collected data on clinicopathological features, computed tomography (CT) imaging histological features, RT dosimetric parameters, and inflammatory markers of patients to develop and validate a non-invasive and personalized predictive model for the occurrence of RE in patients with NSCLC who underwent RT after immunochemotherapy (i.e., who had received ICIs prior to RT, ICI-RT).

## 2 Methods

### 2.1 Patients

**Figure 1:**
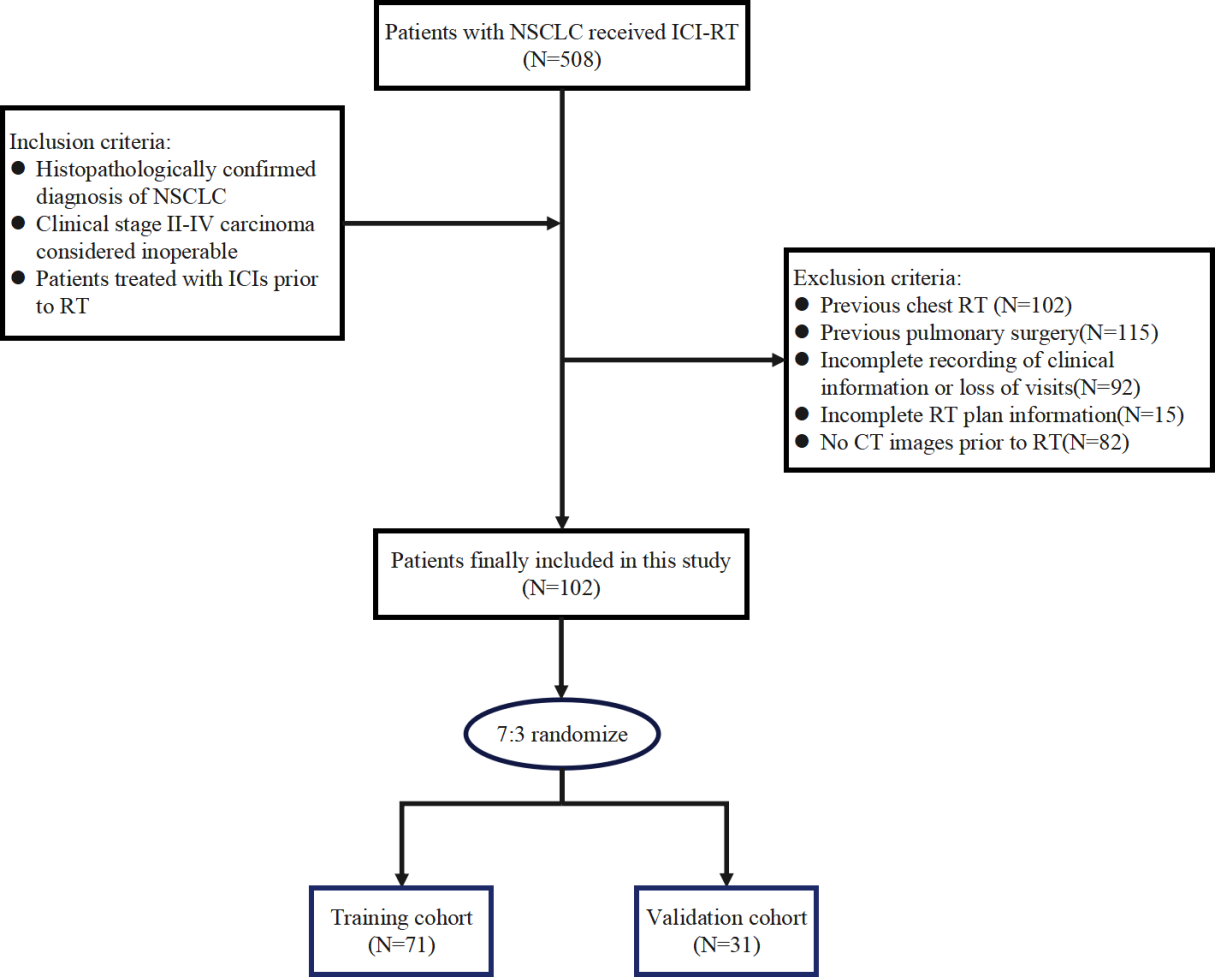
Flow chart for patient inclusion.

The data of patients with NSCLC who underwent ICI-RT from April 2021 to September 2023 at the Shandong Cancer Hospital and Institute were retrospectively analyzed. The inclusion criteria were as follows: (a) histopathologically confirmed diagnosis of NSCLC, which included squamous cell carcinoma, adenocarcinoma, and other types of NSCLC, such as large cell carcinoma; (b) clinical stage II–IV carcinoma considered inoperable; and (c) patients treated with ICIs prior to RT. The exclusion criteria were as follows: (a) previous chest RT and (b) incomplete recording of clinical information or loss of visits. A total of 102 patients were eligible for enrollment and randomly assigned to a training (n = 71) or a validation (n = 31) set in a 7:3 ratio, as shown in **Figure 1**. This study was approved by the Ethics Committee of the Shandong Cancer Hospital and Institute (approval number: SDTHEC2022009020), which waived the requirement for participant informed consent considering the retrospective nature of the study. The study was conducted in accordance with the ethical guidelines outlined in the Declaration of Helsinki.

### 2.2 Treatment regimen and dosimetric data

All patients underwent intensity-modulated radiation therapy or three-dimensional conformal radiation therapy. The RT dose was 36–70 Gy fractions, 5 days per week, 1 time per day. All patients underwent CT scanning with a 3 mm slice thickness using a Philips 16-slice Brilliance large-aperture CT scanner (Philips Medical Systems). The CT images were imported into the Eclipse 16.1 (Varian) planning system for target area and organ at risk outlining. The gross tumor volume (GTV) was defined as the primary tumor and metastatic lymph nodes visible on imaging. The clinical target volume (CTV) was defined as microscopically visible tumor microfoci outside the GTV. CTV margins were 0.8 cm beyond the GTV for adenocarcinoma and 0.6 cm beyond the GTV for squamous cell cancer, including the drainage area of the positive lymph nodes. The planning target volume was 0.5–1 cm outside the CTV owing to various errors.

Dosimetric parameters such as D_max_, MED, V_20_, V_30_, V_40_, V_50_, and V_60_ were extracted from dose-volume histograms of the Eclipse 16.1 planning system. V_n_ was defined as the percentage of the total esophageal volume irradiated with doses exceeding n Gy.

All patients underwent ICIs prior to RT, and chemotherapy could be administered concurrently with ICIs. Immunotherapy regimens were 1–8 cycles of programmed cell death-1 (PD-1) inhibitor administered intravenously (pembrolizumab at a dose of 200 mg, camrelizumab at a dose of 200 mg, tislelizumab at a dose of 200 mg, or sintilimab at a dose of 200 mg) every 3 weeks or programmed cell death-ligand 1 (PD-L1) inhibitor (atezolizumab at a dose of 1200 mg) every 3 weeks. Chemotherapy regimens consisted mainly of platinum drugs combined with pemetrexed or paclitaxel/albumin paclitaxel intravenous infusion therapy. The former consisted of platinum agents (carboplatin area under the curve 5–6 or cisplatin 75 mg/m^2^, day 1) and pemetrexed 500 mg/m^2^, day 1; the latter chemotherapy regimen consisted of paclitaxel 135–175 mg/m^2^, day 1 or albumin paclitaxel 125 mg/m^2^, days 1 and 8 in combination with platinum agents. Patients underwent chemotherapy every 3 weeks for an average of 4 cycles, with the dose of chemotherapy adjusted according to patient tolerance.

### 2.3 Toxicity assessment

Patients were assessed weekly for toxicities during RT and followed up monthly for 6 months after the end of RT. The diagnosis of RE was based on a combination of clinical presentation, upper gastrointestinal barium meal, hematology test results, and endoscopy findings. The RE classification was based on the Common Terminology Criteria for Adverse Events v5.0. as follows: (a) Grade 1: asymptomatic, clinical or diagnostic findings only, no treatment required; (b) Grade 2: symptomatic, altered feeding/swallowing, need for nutritional supplementation by mouth; (c) Grade 3: severe alteration of feeding/swallowing, need for nasogastric feeding, total gastrointestinal parenteral nutrition, or hospitalization; (d) Grade 4: life-threatening, need for urgent surgical intervention; and (e) Grade 5: death.

### 2.4 Radiomic feature extraction

We analyzed the CT scans obtained from the enrolled patients before the RT. If multiple CT images were available, the most recent CT images before commencement of RT were used. CT images in DICOM format were extracted from a PACS system. All tumor target areas were manually depicted layer-by-layer by a radiation oncologist using a 3D Slicer (version 5.2.1). The regions of interest (ROI) were then confirmed by another clinician experienced in chest CT analysis. All features were extracted using the open source software package Pyradiomics in 3D Slicer. To reduce the variation across different patient images, Z-score normalization was performed on all data as a preprocessing step. To reduce any type of bias or overfitting caused by too many features, features with high repeatability and stability were first screened by calculating the intraclass correlation coefficient (ICC). In this study, the features were screened using ICC > 0.9 as the criterion. After the initial screening of features with high repeatability, these features were further downscaled and screened for key features using least absolute shrinkage and selection operator regression (17, 18). Finally, the selected features and corresponding weighting coefficients were linearly combined to create a radiomic score (Rad-score) for each patient (19).

### 2.5 Model construction and evaluation

The point with the largest Youden’s index was determined as the optimal cutoff value for each parameter using receiver operating characteristic (ROC) curves. Univariate logistic regression was used to analyze the correlation between clinicopathological characteristics, RT dosimetry parameters, inflammatory indicators, Rad-score, and RE in the training cohort. Factors with *p* < 0.05 were included in the multivariate logistic regression analysis to screen for independent risk factors. Next, a nomogram was constructed using factors with significant predictive values derived from the multivariate analysis. Finally, the predictive performance of the nomogram model for RE was evaluated using the area under the ROC curve (AUC), calibration curves, and decision curves in the training and validation cohorts.

### 2.6 Statistical analysis

Continuous variables were compared using independent sample t-tests or rank-sum tests, whereas categorical variables were compared using chi-square or Fisher’s exact tests. Univariate and multivariate logistic regression analyses were performed to identify independent risk factors for RE. Spearman rank correlation coefficients were used to assess the relationship between dose parameters. All tests were two-sided, and *p* < 0.05 was considered statistically significant. All data analyses and graphing were performed using SPSS software (version 25.0; IBM Corp.) and R software (version 4.3.2.).

## 3 Results

### 3.1 Patient characteristics and incidence of RE

A total of 102 patients with NSCLC treated with ICI-RT participated in this study. Of these, 88 (86.3%) were male and 43 (42.2%) aged ≤60 years. The tumors were located in the left and right lung of 39 (38.2%) and 63 (61.8%) patients, respectively. More than half of the patients had squamous cell carcinoma of the pathologic type. Seventy-one patients were included in the training cohort and 31 were included in the validation cohorts, respectively. **Table 1** summarizes the baseline characteristics of the training and validation cohorts.

**Table 1:** Basic clinical information of patients in the training and validation cohorts.

Characteristics	Training (n = 71)	Validation (n = 31)	χ²	*p-value*
Sex (%)				
Female	9 (12.68)	5 (16.13)	0.02	0.878
Male	62 (87.32)	26 (83.87)		
Age (%)				
≤60	30 (42.25)	13 (41.94)	0.00	0.976
>60	41 (57.75)	18 (58.06)		
Pathology (%)				
Squamous cell carcinoma	39 (54.93)	16 (51.61)	0.10	0.757
Adenocarcinoma	32 (45.07)	15 (48.39)		
Clinical stage (%)				
II-III	30 (42.25)	15 (48.39)	0.33	0.566
IV	41 (57.75)	16 (51.61)		
Location (%)				
Right	44 (61.97)	19 (61.29)	0.00	0.974
Left	27 (38.03)	12 (38.71)		
KPS (%)				
≥90	48 (67.61)	21 (67.74)	0.00	0.989
<90	23 (32.39)	10 (32.26)		
Number of treatment cycles (%)				
≤2	22 (30.99)	14 (45.16)	1.90	0.168
>2	49 (69.01)	17 (54.84)		
T stage (%)				
1	9 (12.68)	3 (9.68)	0.82	0.844
2	20 (28.17)	8 (25.81)		
3	13 (18.31)	8 (25.81)		
4	29 (40.85)	12 (38.71)		
N stage (%)				
0	7 (9.86)	2 (6.45)	4.58	0.205
1	14 (19.72)	4 (12.90)		
2	19 (26.76)	15 (48.39)		
3	31 (43.66)	10 (32.26)		
Immunotherapy drugs (%)				
PD-L1	2 (2.82)	2 (6.45)	0.10	0.753
PD-1	69 (97.18)	29 (93.55)		

KPS, Karnofsky performance status; PD-L1, programmed cell death-ligand 1; PD-1, programmed cell death-1.

All patients had undergone prior treatment with PD-1 (96.1%) or PD-L1 (3.9%) inhibitors, and the median time between immunotherapy and initiation of RT was 17 days (IQR, 5–28). The median time interval from RT initiation to RE occurrence was 18.5 days (IQR, 13.25–24.25). In total, 64 (62.7%) patients did not develop RE, whereas 38 (37.3%) developed RE. Of these, 30 (29.4%) had grade 2 RE, 1 (1.0%) had grade 3 RE, and no patients had grade 4–5 RE. RE occurred in 28 (39.4%) and 10 (32.3%) patients in the training and validation cohorts, respectively. **Table 2** lists the pre-RT inflammatory indices and RT dosimetric parameters of patients in the training and validation cohorts. No significant differences were observed between the cohorts with respect to the clinicopathological features, dosimetric parameters, and inflammatory indices (*p* > 0.05).

**Table 2:** Inflammatory indicators and radiotherapy parameters of patients in the training and validation cohorts.

Characteristics	Training (n = 71)	Validation (n = 31)	χ²	*p-value*
NLR				
≤3.3	49 (69.01)	24 (77.42)	0.75	0.387
>3.3	22 (30.99)	7 (22.58)		
LMR				
≤3.8	51 (71.83)	23 (74.19)	1.85	0.828
>3.8	20 (28.17)	8 (25.81)		
PLR				
≤178.0	42 (59.15)	18 (58.06)	0.01	0.918
>178.0	29 (40.85)	13 (41.94)		
SII				
≤703.4	48 (67.61)	21 (67.74)	0.00	0.989
>703.4	23 (32.39)	10 (32.26)		
PAR				
≤4.0	30 (42.25)	9 (29.03)	1.60	0.206
>4.0	41 (57.75)	22 (70.97)		
D_max_				
≤58.4	40 (56.34)	14 (45.16)	1.08	0.298
>58.4	31 (43.66)	17 (54.84)		
MED				
≤13.3	43 (60.56)	13 (41.94)	3.02	0.082
>13.3	28 (39.44)	18 (58.06)		
V_20_				
≤22.3	31 (43.66)	10 (32.26)	1.17	0.280
>22.3	40 (56.34)	21 (67.74)		
V_30_				
≤15.9	34 (47.89)	11 (35.48)	1.35	0.246
>15.9	37 (52.11)	20 (64.52)		
V_40_				
≤14.1	42 (59.15)	17 (54.84)	0.16	0.685
>14.1	29 (40.85)	14 (45.16)		
V_50_				
≤11.3	47 (66.20)	19 (61.29)	0.23	0.633
>11.3	24 (33.80)	12 (38.71)		
V_60_				
≤0	28 (39.44)	18 (58.06)	3.02	0.082
>0	43 (60.56)	13 (41.94)		

NLR, neutrophil–lymphocyte ratio; LMR, lymphocyte–monocyte ratio; PLR, platelet–lymphocyte ratio; SII, systemic immunoinflammatory index; PAR, platelet–albumin ratio; D_max_, maximum dose; MED, mean esophagus dose; V_20_, percent of esophagus volume receiving ≥ 20 Gy; V_30_, percent of esophagus volume receiving ≥ 30 Gy; V_40_, percent of esophagus volume receiving ≥ 40 Gy; V_50_, percent of esophagus volume receiving ≥ 50 Gy; V_60_, percent of esophagus volume receiving ≥ 60 Gy.

### 3.2 Rad-score construction

**Figure 2:**
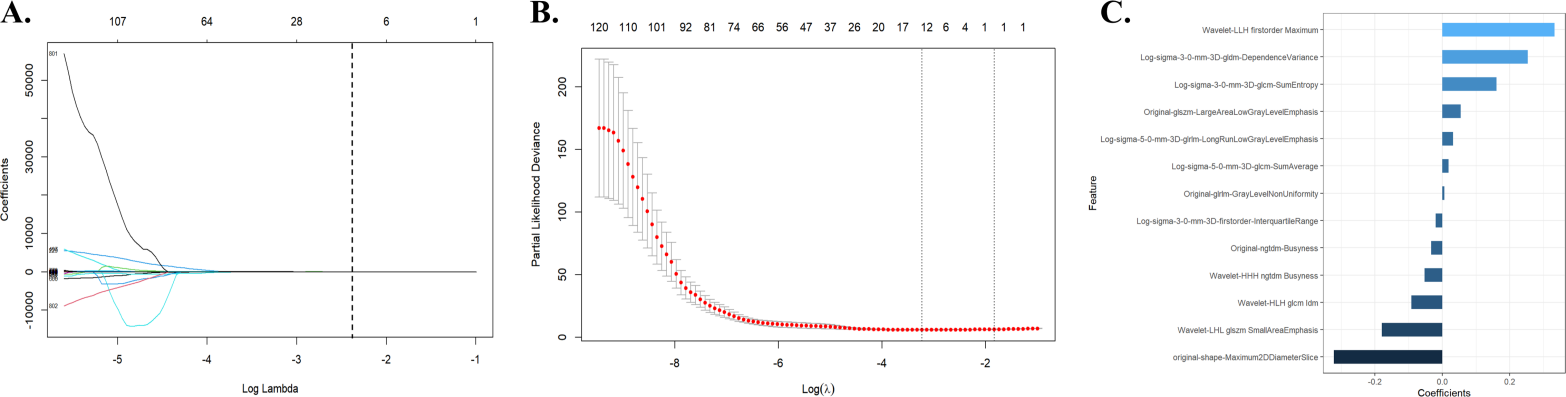
Radiomic feature selection using the LASSO regression model. **(A)** The LASSO regression model identified radiomic features with nonzero coefficients predicting RE. **(B)** Distribution of LASSO regression coefficients for predicting radiomic characteristics of RE. **(C)** Optimal combination of radiomic features and their correlation coefficients for predicting RE. LASSO, least absolute shrinkage and selection operator; RE, radiation esophagitis.


A total of 991 features were extracted from each patient’s ROI using the open-source package Pyradiomics in 3D Slicer software. The extracted radiomics features included shape features, first-order statistical features, gray level co-occurrence matrix, gray level dependence matrix, gray level run-length matrix, gray level size zone matrix, neighbor gray tone difference matrix, and wavelet features. The meaning of these features has been previously described (20), and details are available at https://pyradiomics.readthedocs.io/en/latest/. A total of 13 radiomic features that were most valuable for predicting RE were screened (**Figure 2**). The Rad-score was derived from the linear combinations of the selected key features and corresponding weighting coefficients, as follows:

$$\begin{array}{l} \text{Rad-score}=-0.321496486039533\times \text{original}-\text{shape}\\ -\text{Maximum}2\text{DDiameterSlic}e\\ +-0.179385553956634\times \text{Wavelet}-\text{LHL glszm SmallAreaEmphasis}\\ +-0.091388948744633\times \text{Wavelet}-\text{HLH glcm Idm}\\ +-0.052823747597495\times \text{Wavelet}-\text{HHH ngtdm Busyness}\\ +-0.033454057057413\times \text{Original}-\text{ngtdm}-\text{Busyness}\\ +-0.019905495717509\times \text{Long}-\text{sigma}-3-0-\text{mm}-3\text{D}-\text{firstorder}\\ -\text{InterquartileRange}\\ +0.006413584851584\times \text{Original}-\text{glrlm}-\text{GrayLevelNonUniformity}\\ +0.019058449571357\times \text{Log}-\text{sigma}-5-0-\text{mm}-3\text{D}-\text{glcm}\\ -\text{SumAverage}\\ +0.032345439587447\times \text{Log}-\text{sigma}-5-0-\text{mm}-3\text{D}-\text{glrlm}\\ -\text{LongRunLowGrayLevelEmphasis}\\ +0.055888333774646\times \text{Original}-\text{glszm}\\ -\text{LargeAreaLowGrayLevelEmphasis}\\ +0.161459645775567\times \text{Log}-\text{sigma}-3-0-\text{mm}-3\text{D}-\text{glcm}\\ -\text{SumEntropy}\\ +0.254976457754456\times \text{Log}-\text{sigma}-3-0-\text{mm}-3\text{D}-\text{gldm}\\ -\text{DependenceVariance}\\ +0.333814752875522\times \text{Wavelet}-\text{LLH firstorder Maximum} \end{array}$$

### 3.3 Univariate and multivariate analyses in the training cohort

The univariate logistic regression analysis of the training cohort revealed that left location (*p* = 0.017), Karnofsky performance status (KPS) < 90 (*p* = 0.008), platelet-albumin ratio (PAR) >4.0 (*p* = 0.022), D_max_ > 58.4 Gy (*p* < 0.001), MED > 13.3 Gy (*p* = 0.001), V_20_ > 22.3% (*p* =0.041), V_30_ > 15.9% (*p* = 0.032), V_60_ > 0 (*p* = 0.032), and Rad-score (*p* < 0.001) were potential risk factors for the development of RE (**Table 3**).

**Table 3:** Univariate and multivariate analysis of clinical and radiomic characteristics for RE prediction.

Univariate analysis			Multivariate analysis		
Characteristics	OR (95% CI)	*p-value*	Regression coefficient	OR (95% CI)	*p-value*
Sex					
Female	0.93 (0.79–0.90)	0.898			
Male	Ref				
Age					
≤60	Ref				
>60	0.50 (0.22–1.14)	0.101			
Pathology					
Squamous cell carcinoma	0.92 (0.41–2.06)	0.840			
Adenocarcinoma	Ref				
Clinical stage					
II-III	0.58 (0.26–1.30)	0.184			
IV	Ref				
Location					
Right	Ref		Ref		
Left	2.73 (1.20–6.25)	0.017	1.22	2.72 (0.32–11.48)	0.335
KPS					
≥90	0.26 (0.09–0.70)	0.008	-1.87	0.11 (0.02–1.45)	0.117
<90	Ref		Ref		
Number of treatment cycles					
≤2	0.76 (0.56–0.84)	0.184			
>2	Ref				
T					
1	Ref				
2	1.06 (1.02–2.45)	0.941			
3	1.06 (1.01–3.52)	0.930			
4	1.65 (1.40–2.51)	0.327			
N					
0	Ref				
1	1.23 (1.13–2.12)	0.581			
2	1.77 (1.33–2.44)	0.123			
3	1.01 (1.00–1.32)	0.980			
Immunotherapy drugs					
PD-L1	1.62 (0.23–2.76)	0.595			
PD-1	Ref				
Total dose					
≤60	0.62 (0.27–1.41)	0.256			
>60	Ref				
NLR					
≤3.3	Ref				
>3.3	1.28 (0.53–3.08)	0.587			
LMR					
≤3.8	Ref				
>3.8	1.46 (0.58–3.64)	0.423			
PLR					
≤178.0	Ref				
>178.0	1.50 (0.67–3.38)	0.329			
SII					
≤703.4	Ref				
>703.4	2.01 (0.86–4.70)	0.107			
PAR					
≤4.0	Ref		Ref		
>4.0	2.84 (1.16–6.96)	0.022	-0.44	1.75 (1.15–6.33)	0.733
D_max_					
≤58.4	0.06 (0.02–0.18)	<0.001	-2.44	0.09 (0.02–0.99)	0.044
>58.4	Ref		Ref		
MED					
≤13.3	Ref		Ref		
>13.3	25.89 (8.44–79.40)	0.001	4.22	6.23 (1.98–9.56)	0.027
V_20_					
≤22.3	Ref				
>22.3	1.99 (1.59–10.03)	0.041			
V_30_					
≤15.9	Ref				
>15.9	2.14 (2.04–12.95)	0.032			
V_40_					
≤14.1	Ref				
>14.1	1.37 (1.46–7.80)	0.094			
V_50_					
≤11.3	Ref				
>11.3	1.33 (1.42–7.81)	0.101			
V_60_					
≤0	Ref		Ref		
>0	2.14 (2.04–12.95)	0.032	1.54	1.47 (0.91–3.55)	0.225
Rad-score	0.30 (0.19–0.44)	<0.001	-1.12	0.24 (0.10–0.58)	0.002

RE, radiation esophagitis; OR, odds ratio; CI, confidence interval; KPS, Karnofsky performance status; PD-L1, programmed cell death-ligand 1; PD-1, programmed cell death-1; NLR, neutrophil–lymphocyte ratio; LMR, lymphocyte–monocyte ratio; PLR, platelet–lymphocyte ratio; SII, systemic immunoinflammatory index; PAR, platelet–albumin ratio; D_max_, maximum dose; MED, mean esophagus dose; V20, percent of esophagus volume receiving ≥ 20 Gy; V30, percent of esophagus volume receiving ≥ 30 Gy; V40, percent of esophagus volume receiving ≥ 40 Gy; V50, percent of esophagus volume receiving ≥ 50 Gy; V_60_, percent of esophagus volume receiving ≥ 60 Gy; Rad-score, radiomic score.

**Figure 3:**
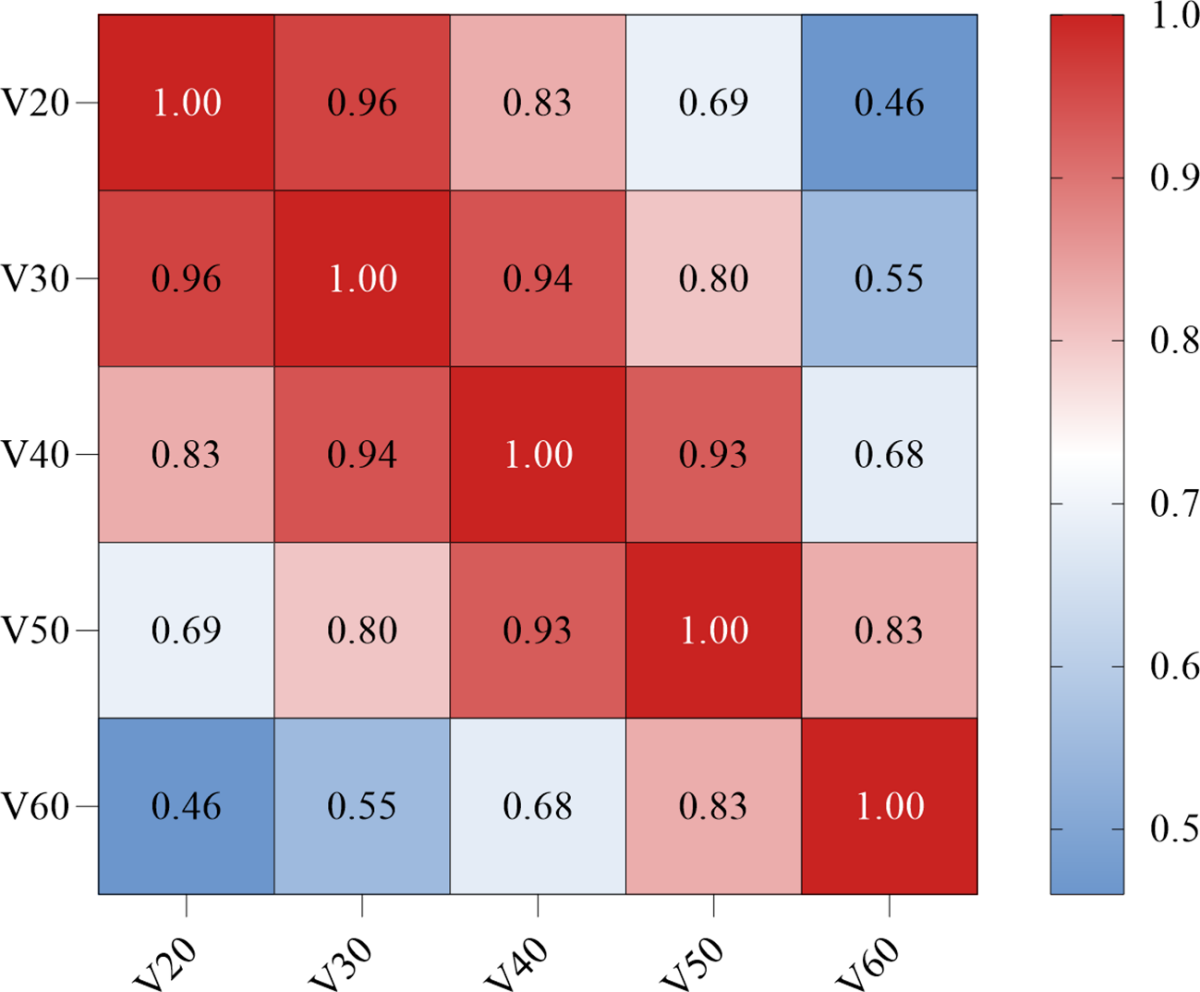
Correlation between the dosimetric parameters Vn (Spearman coefficient). The darker the red color, the stronger the positive correlation. V_20_, percent of esophagus volume receiving ≥ 20 Gy; V_30_, percent of esophagus volume receiving ≥ 30 Gy; V_40_, percent of esophagus volume receiving ≥ 40 Gy; V_50_, percent of esophagus volume receiving ≥ 50 Gy; V_60_, percent of esophagus volume receiving ≥ 60 Gy.

As shown in **Figure 3**, a significant correlation was observed between the Vn parameters. Therefore, left location, KPS < 90, PAR > 4.0, D_max_ > 58.4 Gy, MED > 13.3 Gy, V_60_ > 0, and Rad-score were included in the multivariate logistic regression analysis. The results showed that D_max_ > 58.4 Gy (odds ratio [OR]: 0.09, 95% confidence interval [CI]: 0.02–0.99, *p* = 0.044), MED > 13.3 Gy (OR: 6.23, 95%CI: 1.98–9.56, *p* = 0.027), and Rad-score (OR: 0.24, 95%CI: 0.10–0.58, *p* = 0.002) all maintained significant differences and were independent predictors of RE occurrence (**Table 3**).

### 3.4 Establishment and evaluation of the nomogram

**Figure 4:**
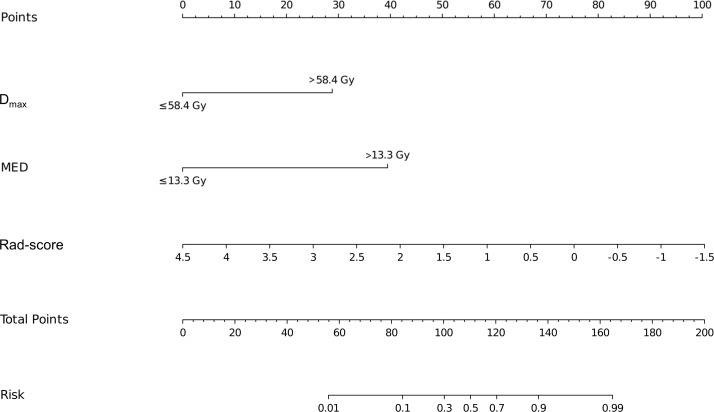
Nomograms used for predicting RE in the training cohort. Nomogram incorporating the Dmax, MED, and Rad-score from patients with NSCLC. RE, radiation esophagitis; Dmax, maximum dose; MED, mean esophageal dose; Rad-score, radiomic score; NSCLC, non-small cell lung cancer.

**Figure 5:**
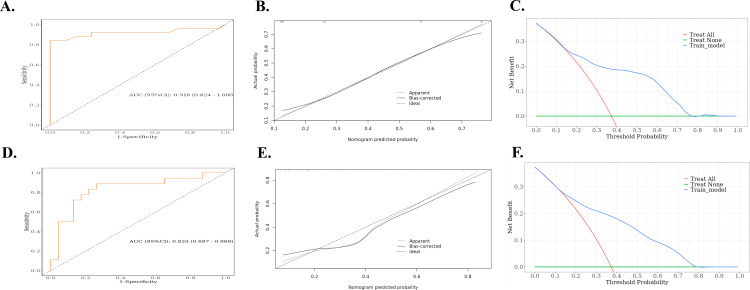
ROC, calibration curves, and decision curves for nomograms predicting RE in the training cohort and validation cohort. **(A)** ROC curves for the nomograms for the training cohort. **(B)** Calibration curves for the nomograms for the training cohort. The x and y axes represent the predicted and actual probabilities, respectively. **(C)** Decision curves for the nomograms for the training cohort. The x and y axes represent the threshold probability and net benefit, respectively. **(D)** ROC curves for the validation cohort nomogram. **(E)** Calibration curves for the validation cohort nomogram. The x and y axes represent the predicted and actual probabilities, respectively. **(F)** Decision curves for the validation cohort nomogram. The x and y axes represent the threshold probability and net benefit, respectively. ROC, receiver operating characteristic; RE, radiation esophagitis.

Based on the results of the multivariate logistic regression analysis, a nomogram was constructed using the identified independent predictors (**Figure 4**). The ROC curves indicated the AUC of the model in the training and validation cohorts to be 0.918 (95%CI: 0.824–1.000) and 0.833 (95%CI: 0.697–0.969), respectively, indicating the good discriminative ability of the model (**Figures 5A, D**). In addition, the predictive performance of the nomogram and three other independent predictors for RE was compared. In the training cohorts, the AUCs based on the MED, D_max_, and Rad-score models were 0.857, 0.797, and 0.684, respectively, and the nomogram model obtained an AUC of 0.918 (**Supplementary Figure 1A**). In the validation cohorts, the AUCs based on the MED, Dmax, and Rad-score models were 0.779, 0.633 and 0.630, respectively, while the AUC of the nomogram model is 0.833 (**Supplementary Figure 1B**). The calibration curves of the training and validation cohorts showed good agreement between the actual and predicted occurrence probabilities of RE (**Figures 5B, E**), and decision curves showed that the nomogram had a positive net benefit for most threshold probabilities, suggesting that the model had satisfactory clinical outcomes (**Figures 5C, F**).

## 4 Discussion

According to the PACIFIC criteria, approximately half of the patients with unresectable NSCLC undergoing cCRT are not eligible for durvalumab treatment (21, 22). RT after induction immunochemotherapy has become a viable alternative treatment strategy for patients with unresectable locally advanced NSCLC (23). However, the combination of ICIs and RT also increases the risk of RE. In this study, a total of 38 (37.3%) patients developed RE; however, the field currently lacks a validated tool to identify patients at high risk of developing RE. To the best of our knowledge, this was the first study to use a predictive model for the development of RE after treatment with a combination of ICIs and RT.

In this study, after multivariate analysis, D_max_ > 58.4 Gy, MED > 13.3 Gy, and radiomic features were identified as independent risk factors for the development of RE following RT after immunochemotherapy. The AUC values were 0.918 and 0.833 for the training and validation cohorts, respectively, indicating good discrimination.

Our study revealed D_max_ and MED to be the independent risk factors for RE, and the optimal thresholds for D_max_ and MED in ICI-RT were 58.4 Gy and 13.3 Gy, respectively. Huang et al. retrospectively analyzed 193 patients with NSCLC undergoing cCRT, multivariate analysis showed that MED and V_10_–V_60_ were significantly correlated with RE and that the MED model was the best fitted model compared with models with other parameters (24). Belderbos et al. evaluated 156 patients with inoperable or locally advanced NSCLC to analyze the relationship between clinical and dosimetric parameters and acute esophageal toxicity. The results showed that the most important clinical parameter predicting acute esophageal toxicity was cCRT, and among the dosimetric parameters, V_35_ was the strongest predictor of grade ≥2 acute esophageal toxicity (25). Notably, Kim et al. showed that V_60_ was significantly associated with the occurrence of grade 3 and higher RE (26). Furthermore, a meta-analysis by Palma et al. based on data from 1082 patients treated with cCRT revealed that although D_max_ was significant in univariate analysis, it was not an independent risk factor. Only V_60_ was the best predictor of grade 2 and 3 RE with good calibration and discrimination (27). This difference can be attributed to the fact that previous studies were based on the direct effects of RT on tumors and normal tissues; however, the addition of ICIs strengthens the antitumor immune response by inducing lymphocyte differentiation and upregulating cytokine and autoantibody levels, resulting in excessive cytokine release and increased immune cell infiltration, which amplifies the inflammatory response in irradiated normal tissues (28). In addition, the antitumor effect on the body is enhanced after the application of ICIs, and the accumulation of self-DNA released from dead tumor cells can trigger the cGAS–STING signaling pathway, which induces the production of interferon and inflammatory cytokines and ultimately triggers an inflammatory response (29).

Artificial intelligence methods to extract tumor information and build machine models have been applied to tumor lymph node metastasis, tumor clinicopathological grading, and T staging (13). Moreover, quantifying tumor heterogeneity is also possible using artificial intelligence, which plays an important role in personalized prediction. Zheng et al. included 161 patients with locally advanced NSCLC treated with RT and developed a model to predict grade ≥2 acute RE based on multi-omics features, including imaging and dosimetry. Multi-omics features exhibited similar predictive properties as radiomics features; however, the separate predictive properties of dosimetry features and clinical factors were limited (30). Xie et al. combined deep learning, radiomics, and dosimetry features to predict RE in patients with esophageal cancer undergoing volumetric modulated arc therapy, and the combination of various feature extraction methods improved the accuracy of RE prediction (31). In addition, radiomics exhibit good efficacy in predicting RT-associated lung injury (32, 33). Additional studies have shown that the use of complex static step and shoot technique can reduce radiotherapy-related toxicity by keeping the organs at risk dose within the limits of quantitative analyses of normal tissue effects in the clinic (34). In this study, we selected the 13 radiomics features that were most valuable for predicting RE. The Rad-score was obtained by weighting them according to their respective coefficients and were statistically different in multivariate analyses. These findings support the potential of imaging histology in predicting the occurrence of RE in patients with NSCLC treated with ICI-RT. Internal validation was performed to validate the accuracy of our prediction model. The AUC of the ROC curve, the calibration curve, and the decision curve indicated that the model had good discriminatory power and clinical effectiveness. In the modern era of personalized medicine, integrated multi-omics approaches improve diagnostic accuracy and predictive precision, and this study integrates radiomics, dosimetry, and clinical factors to predict the risk of RE in patients with NSCLC treated with ICI-RT. Our findings provide a new direction for individualized decision-making and the prediction of adverse effects of RT for NSCLC.

Our study also has some limitations. First, avoiding selection bias was difficult owing to the retrospective nature of this study; therefore, further prospective studies on RE are required to validate these results. Second, this was a single-center study, and although internal validation indicated an excellent AUC of the current predictive model, the results would have been more convincing with external validation. Third, the sample size of the present study was relatively small, and future studies should include data from more research centers and larger population samples.

In summary, D_max_ > 58.4 Gy, MED > 13.3 Gy, and Rad-score were independent predictors of RE occurrence in patients with NSCLC treated with ICI-RT. These variables were used to develop and validate a novel nomogram for early screening of patients with NSCLC treated with ICI-RT. The predictive model developed in this study can be used to identify patients who are at increased risk of developing RE during RT.

## Data Availability

The raw data supporting the conclusions of this article will be made available by the authors, without undue reservation.

## Supplementary material

Supplementary Figure 1 ROC curves for single model prediction of RE. **(A)** ROC curves for single model in the training cohort. **(B)** ROC curves for single model in the validation cohort. ROC, receiver operating characteristic; Dmax, maximum dose; MED, mean esophageal dose; Rad-score, radiomic score; AUC, area under the ROC curve; RE, radiation esophagitis.
