# Supplementary figures and images for: Radiomic and dosimetric parameter-based nomogram predicts radiation esophagitis in patients with non-small cell lung cancer undergoing combined immunotherapy and radiotherapy
Source: Front Oncol. 2024 Dec 18;14:1490348. doi: 10.3389/fonc.2024.1490348 (PMC11688372; doi:10.3389/fonc.2024.1490348)

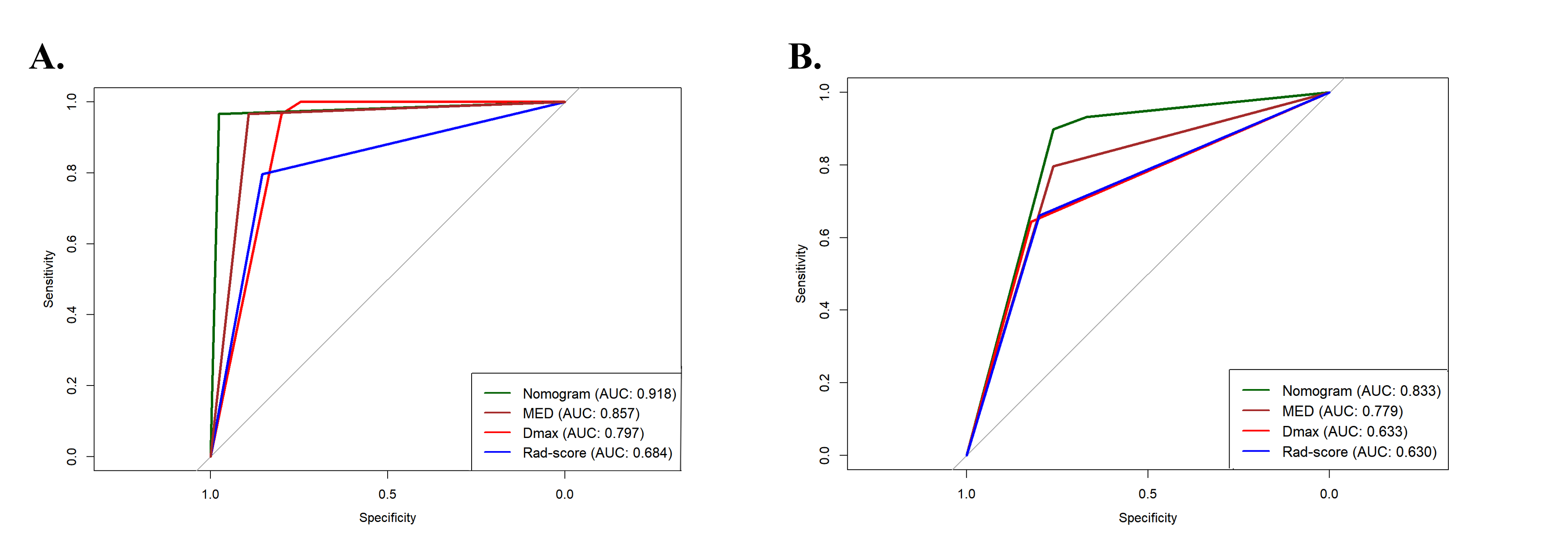

Supplement: Supplementary Figure 1 — ROC curves for single model prediction of RE. (A) ROC curves for single model in the training cohort. (B) ROC curves for single model in the validation cohort. ROC, receiver operating characteristic; Dmax, maximum dose; MED, mean esophageal dose; Rad-score, radiomic score; AUC, area under the ROC curve; RE, radiation esophagitis. [file Image1.tif]
